# Are we there yet?

**DOI:** 10.7554/eLife.49202

**Published:** 2019-06-14

**Authors:** Nicole Swann

**Affiliations:** Department of Human PhysiologyUniversity of OregonEugeneUnited States

**Keywords:** scientist and parent, diversity, careers in science, family friendly policies, conferences, women in science

## Abstract

Traveling to conferences with children presents a number of logistical and financial challenges.

I am an assistant professor and mother of a two-year-old. We recently returned from our third conference together. Despite enormous support from my partner and family, each time I come back from a conference with my child I wonder if it was worth all the stress. Last year I joined a working group of mothers in science and we wrote a piece with suggestions for how conferences can better support parents, especially women (Calisi and A Working Group of Mothers in Science, 2018). Here, I provide a bit more context for what some of the logistical and financial challenges are, and insight into what considerations an academic parent is likely to need to make when traveling with their child or children. Elsewhere, Dr Rebecca Calisi Rodríguez has eloquently discussed the physical, social and emotional challenges involved (Calisi, 2018a; Calisi, 2018b).

## The decision – should you go, and should you bring your children?

Deciding whether or not to attend a conference always involves a careful weighing of options. Does the chance to disseminate your research, get feedback, meet colleagues, start collaborations and engage in networking outweigh the time away and cost? Recently, this discussion has broadened to include the environmental cost of air travel, which has also been causing scientists to reconsider conference travel (Langin, 2019). Choosing whether to attend a conference can be particularly challenging for early-career investigators. Gaining visibility is critical, with some institutions even requiring conference attendance for promotion, but conference travel may mean dipping into a limited start-up fund or spending time away from a fledgling laboratory. If you are a parent, the decision is even more complicated.

If you decide to attend a conference, the next decision you need to make is if you will bring your child(ren) or leave them at home. For some parents, especially ones who are not primary caregivers or who have family or other caregivers nearby, leaving children at home may be the most obvious and easiest choice. For others, including single parents, lactating parents, parents of a child with a disability, parents who share caregiving with a working partner, or whose partner is attending the same conference, leaving children at home may be challenging or impossible. Furthermore, some parents simply do not want to spend much time away from their children. It is important to remember that only certain academics, most often women, have to make this choice, so making either option as easy as possible is a critical step towards inclusivity – which in turn, leads to better science (Nielsen et al., 2017).

**Figure 1:**
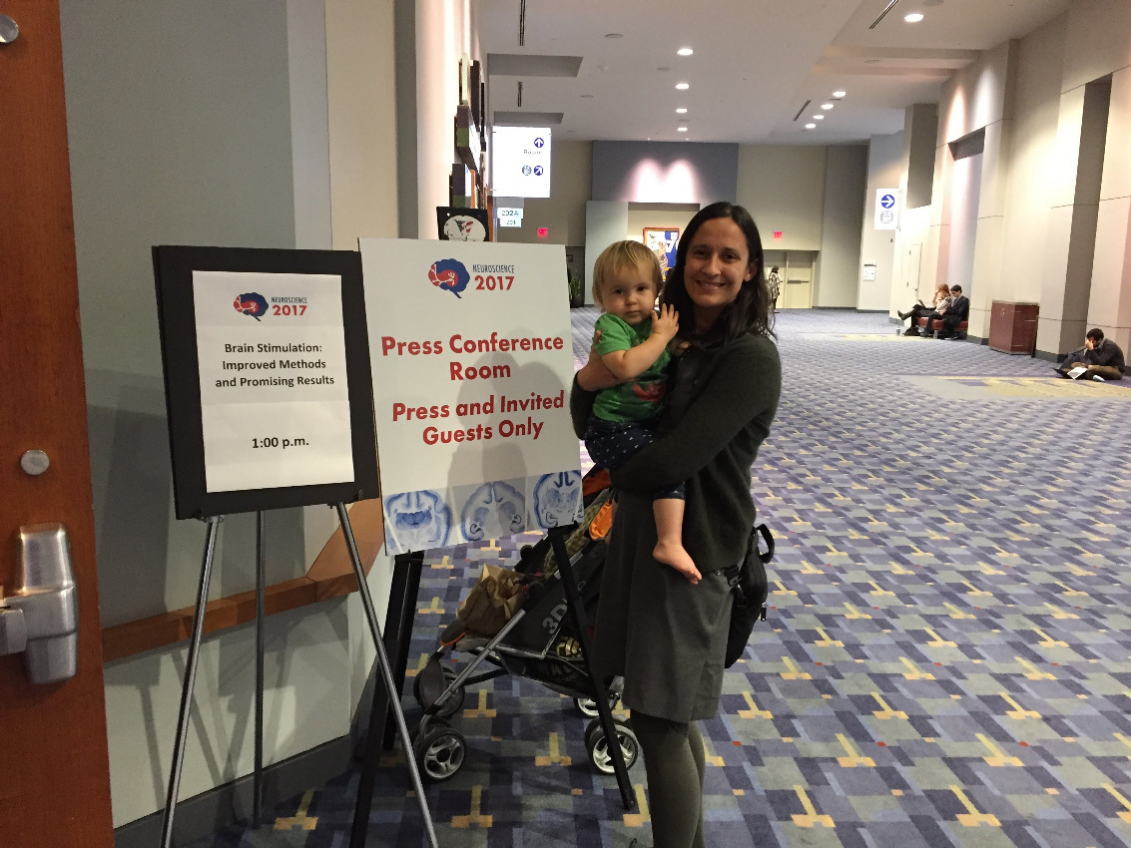
Nicole Swann with her daughter at Neuroscience 2017 in Washington, DC.

IMAGE CREDIT: Ian Greenhouse.

## Logistics of traveling with a child

If you are bringing your child to a conference, the basic logistics that everyone has to deal with (flights, hotel, transportation and meals) become more complicated, and you also have to worry about childcare. Below I discuss some considerations for each.

### Childcare

For conferences that are child-friendly, it may be possible to wear or stroll young infants through the conference center, and older children may be able to entertain themselves quietly during presentations. However, this is not practical for all children, and many parents find that they need to arrange childcare for all or part of the conference. The on-site childcare provided at some conferences can be a great option. Unfortunately, even when available, this service often does not extend into evenings, meaning you will have to skip later sessions and dinners. It can also be expensive (Langin, 2018), and in many cases, funders will not allow you to claim back this cost (Together Science Can, 2018). At a conference I recently attended childcare was $100/day per child – well over the price of conference registration.

The other option is to bring a caregiver with you, perhaps a partner, family member, babysitter or nanny. (I have done this with my daughter.) Of course, this only works if you are lucky enough to have such a person who can accompany you. It also means that the caregiver’s travel costs will have to be paid for, often by you.

### Flights

If your child is under two, you may be able to avoid buying an additional ticket by holding them on your lap (though this is not free for all airlines). However, this does come with drawbacks: say goodbye to finishing your presentation on the plane. Otherwise, you will need to buy a ticket for your child (or children), as well as for any caregivers who are traveling with you – and in most cases, you will not be able to claim these costs back (Together Science Can, 2018).

Bringing a child also often involves additional schedule considerations. You may want to avoid tight connections and very early or very late flights, or want to try to time flights with naps. This may mean that you end up spending more money to get the flights you need.

### Transport at destination

For cities with good public transport, taking your child on buses, trains or subways may be a fun adventure and likely would not add a huge expense. Cities that do not have reliable public transport are more difficult. You may be forced to choose between renting a car (which some institutions may not reimburse) or taking taxis/Ubers/Lyfts. Both of these options might also involve the challenge of trying to install a car seat in an unfamiliar vehicle (perhaps multiple times).

### Food

For some children, dining out exclusively may be a viable option, but for others it can be a recipe for disaster. In any case, eating out frequently can get expensive. Many children also require a seemingly endless supply of snacks, which may require access to a refrigerator or kitchenette.

### Accommodation

Many conferences have a designated conference hotel, which is normally in the most convenient location and may allow you to drop in and out of sessions. However, standard hotel rooms can be small and painfully boring to spend long periods of time in, and the single-room layout may leave you sitting in the dark for hours after your child goes to sleep. This can be particularly frustrating if you know you are missing out on networking events, collaborator meetings or social gatherings.

All this may lead you to consider staying at an extended stay hotel or a condo/apartment/Airbnb, which typically include a kitchen/kitchenette, a separate living space, and perhaps a second bedroom for a caregiver. This extra space may even allow a post-bedtime meeting or social meet-up, if your colleagues are willing to come to you. However, there are negatives too. You may end up based far away from the conference center, and you may run into trouble with reimbursement. For example, some universities do not allow bookings through websites like Airbnb or require employees to use approved hotels. In other cases, there are established per diem hotel rates. For instance, many US universities follow governmental per diem guidelines (which are waived only for designated conference hotels). These rates are often impractically low, especially for expensive cities.

## The need for more flexibility

I do not want to give the impression that attending conferences with your child (or children) is all negative. You get to experience new places with your child, give them a taste of your awesome job, and help normalize parenthood in academia. However, for many parents, choosing to attend conferences can feel like giving much more (financially and otherwise) for what will most likely be a watered-down version of the event.

It is easy to see why some parents may decide to just skip conference travel altogether, especially when many academic parents are early-career researchers, who often have limited financial resources. In short, the cost and flexibility required for traveling with children is incompatible with the rigid guidelines for claiming back expenses put in place by the universities and funders. This stacks the deck against academic parents, and disproportionately affects women, since biology, cultural and societal norms mean that they more often have to choose to bring their child with them or not travel at all. These same considerations are further compounded for academics with limited financial resources, meaning that some of the most vulnerable and under-represented groups in academia are most impacted.

The burden should not be on parents to figure out how to survive in a culture that was not built for them

## Suggestions

Besides giving yourself space for self-care and attending conferences that support you, I do not have a game-changing suggestion for parents that will solve the challenges of traveling to conferences with small children. But I don’t think I should have to propose these solutions. The burden should not be on parents to figure out how to survive in a culture that was not built for them. Instead, it is time to change that culture.

Small things can be done to help. Conferences can include free or subsidized childcare and family-friendly spaces (Calisi and A Working Group of Mothers in Science, 2018; Langin, 2018), and universities can provide travel grants to parents to cover the additional costs of work-related travel with children (Bos et al., 2018). Funding agencies can offer similar grants or at least reimburse childcare and the travel costs of children and caregivers. Additionally, rules for allowable expenses could loosen restrictions on hotel reimbursement or means of transport. Finally, universities can reconsider requirements for promotion or tenure, offering flexibility for criteria that rely on travel. Such changes would be relatively inexpensive, but could make conference travel far more achievable for parents, making academia a more welcoming and inclusive place.

## Note

This Feature Article is part of the Scientist and Parent collection.
